# Hierarchical Multimodal Adaptive Fusion (HMAF) Network for Prediction of RGB-D Saliency

**DOI:** 10.1155/2020/8841681

**Published:** 2020-11-20

**Authors:** Ying Lv, Wujie Zhou

**Affiliations:** ^1^School of Information and Electronic Engineering, Zhejiang University of Science & Technology, Hangzhou 310023, China; ^2^College of Information Science and Electronic Engineering, Zhejiang University, Hangzhou 310027, China

## Abstract

Visual saliency prediction for RGB-D images is more challenging than that for their RGB counterparts. Additionally, very few investigations have been undertaken concerning RGB-D-saliency prediction. The proposed study presents a method based on a hierarchical multimodal adaptive fusion (HMAF) network to facilitate end-to-end prediction of RGB-D saliency. In the proposed method, hierarchical (multilevel) multimodal features are first extracted from an RGB image and depth map using a VGG-16-based two-stream network. Subsequently, the most significant hierarchical features of the said RGB image and depth map are predicted using three two-input attention modules. Furthermore, adaptive fusion of saliencies concerning the above-mentioned fused saliency features of different levels (hierarchical fusion saliency features) can be accomplished using a three-input attention module to facilitate high-accuracy RGB-D visual saliency prediction. Comparisons based on the application of the proposed HMAF-based approach against those of other state-of-the-art techniques on two challenging RGB-D datasets demonstrate that the proposed method outperforms other competing approaches consistently by a considerable margin.

## 1. Introduction

Saliency prediction, wherein the objective is to automatically predict what most attracts human attention in view-free scenarios, is a long-standing classical research topic concerning visual-cognition, computer sciences [1, 2], and imaging techniques [3–6]. Saliency models can be broadly classified into two types—(1) salient-object detection and (2) saliency prediction. In the former, the objective is to detect the most conspicuous and eye-catching objects within scenes accurately [7–16], whereas the latter aims at locating a set number of eye-fixation points during the viewing of a scene [17–23]. This study focuses on the second task, namely, saliency prediction.

In the last two decades, numerous saliency prediction methods for RGB images have been significantly improved, and various models have been proposed [17–19]. However, several extant studies [1, 8, 11] reveal that features extracted from two modalities—depth maps and RGB images—complement each other. RGB images contain discriminative visual-appearance information, whereas depth maps include geometric features concerning objects. In depth maps, the interestingness of objects degrades with increasing distance from the camera; i.e., an object located closer to the camera attracts greater attention. RGB-D saliency prediction has attracted increased attention in recent years [24–28], and extant studies have concentrated on the design of depth-induced RGB-D saliency prediction methods [29–31]. In most such methods, fusion methods are inadequate to combine complementary features obtained from an RGB image and depth map. Therefore, substantial room for performance improvement exists.

This study was inspired by the above-mentioned observation. To facilitate the appropriate fusion of features obtained from the RGB image and depth map, a hierarchical multimodal adaptive fusion (HMAF) network for RGB-D saliency prediction is proposed. In the proposed method, three two-input attention modules are adopted to exploit the importance weights of different modalities instead of simply concatenating feature vectors obtained from the two channels. Additionally, a three-input attention module is used to fuse the hierarchical fusion saliency features adaptively, thereby facilitating accurate RGB-D visual saliency prediction. The major contributions of this study to the literature can be summarized as follows:

1. A two-stream network was established to extract hierarchical multimodal features from an RGB image and depth map, where the features are extracted from different levels with different properties.
2. The hierarchical features of two modalities were first adaptively fused using three two-input attention modules. Subsequently, the output hierarchical features were fused by a three-input attention module to facilitate high-accuracy RGB-D visual saliency prediction.
3. To demonstrate the robustness and effectiveness of the proposed HMAF-based network, it has been qualitatively and quantitatively compared against other state-of-the-art techniques via extensive experiments performed on two challenging RGB-D saliency prediction datasets. The results of these comparisons demonstrate that the proposed HMAF-based method outperforms all relevant techniques available to date.

## 2. Proposed Network Architecture

**Figure 1:**
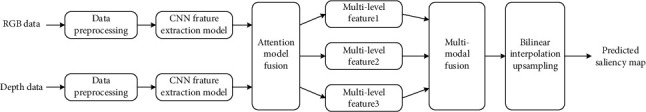
Implementation flow of the proposed method for the prediction of RGB-D saliency.

**Figure 2:**
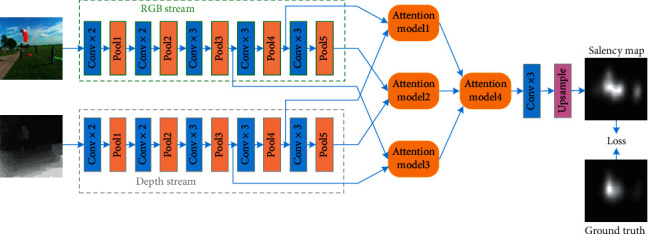
Proposed HMAF.

Figure 1 is a block diagram of the proposed algorithm flow. In this section, we first introduce the pipeline of our HMAF for RGB-D-saliency prediction. We use a two-stream network to extract the hierarchical features of two modalities. Then, three two-input attention modules can adaptively fuse the hierarchical features of the two modalities. Finally, the hierarchical fusion saliency features are adaptively fused by the three-input attention module.  Figure 2 shows the proposed HMAF-based approach.

### 2.1. Hierarchical Multimodal Feature Extraction

Features existing at different levels possess different properties. High-level features contain more semantic information—useful for distinguishing salient areas—but very little spatial and local-context information. In contrast, low-level features contain more spatial information (e.g., textures, edges, and contours). Thus, both high- and low-level features can be considered important and complementary to each other regarding the prediction of visual attention, thereby making multilevel feature extraction even more necessary to achieve high-accuracy saliency prediction.

In this study, VGG-16 [32]—a widely used pretrained network—was considered as the backbone of the two-stream network for extracting hierarchical features from the RGB and depth modalities. However, modifications to the VGG-16 network were affected via the elimination of its fully connected layer. To preserve the relatively large spatial sizes within higher layers, the stride of the last max-pooling layer was decreased, thereby facilitating more accurate saliency prediction. Therefore, the stride of the entire network was reduced to 16 for a fixed input RGB (depth) image size of *M* × *N* pixels. The spatial size of the final feature map was *M*/2^4^  ×  *N*/2^4^. In the proposed study, the output hierarchical features from max-pooling layers Pool3, Pool4, and Pool5 were used to obtain hierarchical fusion saliency feature maps concerning the adaptive multimodal fusion. High-level features can be used to distinguish between different object classes while being less discriminative of objects belonging to the same class. In contrast, low-level features include more spatial information that can be distinguished within the same class of objects, but they are less robust to dramatic changes in appearance. Both high- and low-level features were used in this study to enhance the performance of saliency prediction. Accordingly, in the proposed HMAF-based approach, hierarchical multimodal features were extracted from layers Pool3, Pool4, and Pool5, as shown in Figure 2.

### 2.2. Fusion of Multimodal Features

**Figure 3:**
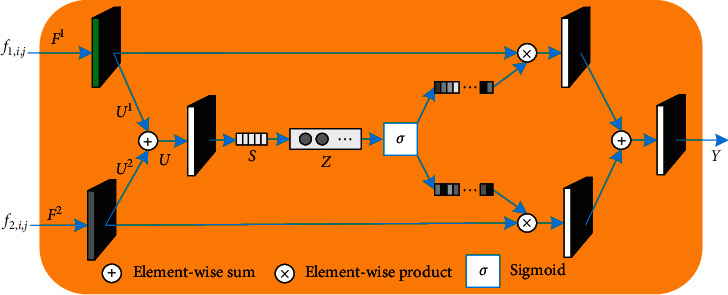
Two-input attention module (attention modules 1, 2, and 3).

Attention modules assign different weights to the features extracted from the two modalities [33]. The two-input attention modules employed in this study comprise three operators—*Transformation*, *Fuse*, and *Select*, as illustrated in Figure 3, where a two-input case is illustrated.

#### 2.2.1. Transformation

Let *f*_*m*, *i*, *j*_ ∈ *ℝ*^*H* × *W* × *C*^ represent the feature maps of *m* modalities (values 1 and 2 of *m* corresponding to the RGB and depth modalities, respectively), wherein *i* can assume the values 1, 2, and 3 to represent the extraction of different level features from max-pooling layers Pool3, Pool4, and Pool5 of VGG-16, respectively, and *j* denotes the spatial position. Two transformations—ℱ^1^: *f*_1, *i*, *j*_ *⟶* *U*^1^ ∈ *ℝ*^*H* × *W* × *C*^ and ℱ^2^: *f*_2, *i*, *j*_ *⟶* *U*^2^ ∈ *ℝ*^*H*^^*×*^^*W*^^*×*^^*C*^—were first performed with a dilated convolution involving a 3 × 3 kernel and dilation size of 2.

#### 2.2.2. Fuse

Transformation results obtained from the two modal streams were subsequently fused through an element-wise summation expressed as

(1) $$\begin{array}{c} U=U^{1}+U^{2}. \end{array}$$

The global information obtained was subsequently embedded using global average pooling to obtain channel-wise features—*S* ∈ ℝ^*C*^. Furthermore, a compact feature *Z* ∈ *ℝ*^*d*×1^ was created to facilitate guidance for precise and adaptive selections. This was accomplished with the use of a fully connected layer, with a decrease in dimensionality for better efficiency, where *Z* = *δ*(*β*(**W***S*)); *β* is the batch normalization; *δ* denotes the rectified linear unit function, and **W** ∈ ℝ^*d*×1^.

#### 2.2.3. Select

According to soft computing techniques [33, 34], a soft attention across streams was used to select different streams guided by the feature Z. In particular, a fully connected layer with a sigmoid operator was applied to stream-wise digits to generate a probability statistical distribution comprising two streams,

(2) $$\begin{array}{c} w_{1,c}=\frac{e^{A_{c}Z}}{e^{A_{c}Z}+e^{B_{c}Z}},\\ w_{2,c}=\frac{e^{B_{c}Z}}{e^{A_{c}Z}+e^{B_{c}Z}}. \end{array}$$

Here, *A*_*c*_ (*B*_*c*_) denotes the *c*^th^ row of *A* (*B*), and *w*_1,*c*_ (*w*_2,*c*_) denotes the *c*^th^ element of *a* (*b*). Additionally, *A*, *B* and *a*, *b* denote the soft attention vectors for *U*^1^ and *U*^2^, respectively. The final output feature map *Y* was obtained by redistributing weights via *w*_1,*c*_ and *w*_2,*c*_ to features corresponding to modalities RGB and depth, respectively. The corresponding equation can be written as

(3) $$\begin{array}{c} Y=w_{1,c}\cdot U^{1}+w_{2,c}\cdot U^{2}. \end{array}$$

**Figure 4:**
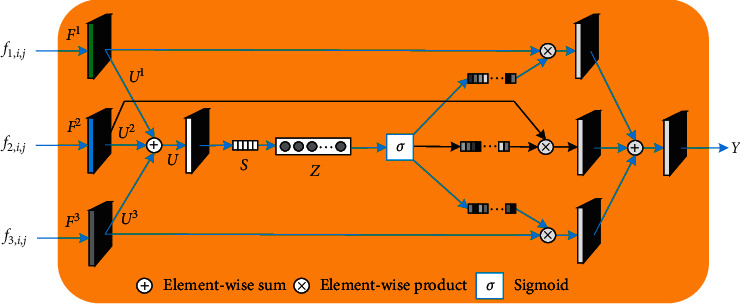
Three-input attention module (attention module 4).

It must be noted that the above equation is only applicable to the two-input case. However, one can easily deduce the corresponding formulae for cases involving more inputs by simply extending equations (1)–(3), and the detailed structure of a three-input case is shown in Figure 4.

### 2.3. Saliency Prediction

Upon completion of the multimodal feature fusion stage, hierarchical fusion saliency features elevated to the same size were obtained via bilinear interpolation. Subsequently, the final fusion result was obtained via the use of a three-input attention module (by extending equations (1)–(3)). As previously mentioned in Section 2.2, the attention module can adaptively assign importance weights to fused saliency features belonging to different levels.

After obtaining the final fusion result, the overall saliency-prediction result was obtained via a combined operation of two convolution layers, a final prediction with a 1 × 1 convolution layer, and a sigmoid activity function. To obtain the same resolution as the input, bilinear interpolation was performed using a factor of four.

### 2.4. Combined Loss Function

In this study, the mean square error (MSE) was used as a baseline reference in combination with the linear correlation coefficient (CC) criteria to train the proposed HMAF network. However, the CC criteria were slightly modified to represent dissimilarities without the need for empirical coefficients. The modified loss was observed to mimic cross-entropy behavior, which is used in visual classification, approaching zero in the absence of any mistakes.

The MSE function is generally used to detect deviations between the predicted value (*P*) and the true value (*Q*) of the model. The smaller the value is, the closer the predicted value is to the real value. Its calculation formula is as follows:

(4) $$\begin{array}{c} \text{MSE}=\frac{1}{N}\sum_{i=1}^{n}{\left\|P-Q\right\|}^{2}, \end{array}$$

where *N* is the number of pixels per image; *i* indicates the *i*^th^ pixel; index *n* ranges from 1 to *N*; *Q* denotes the ground-truth, and *P* represents the predicted saliency map.

The CC criteria are deployed to calculate the linear CC between two distributions, and its value lies within the range [−1, 1], with CC = 1 indicating that the two distributions are correlated and vice versa. The CC function is defined as follows:

(5) $$\begin{array}{c} \text{CC}\left(P,Q\right)=\frac{\sigma \left(P,Q\right)}{\sigma \left(P\right)\times \sigma \left(Q\right)}, \end{array}$$

where *σ* represent the standard deviation of the input.

For the efficient application of root mean square propagation (RMSprop), the CC criteria can be simplified to the form

(6) $$\begin{array}{c} CC^{\prime }\left(P,Q\right)=1-\frac{\sigma \left(P,Q\right)}{\sigma \left(P\right)\times \sigma \left(Q\right)}, \end{array}$$

where *CC′* represents the modified CC criteria. It converts the similarity criteria into dissimilarity criteria, the value of which lies within the range [0, 2]. In this study, a simple summation of *MSE* and *CC′* was considered to formulate the loss function, i.e.,

(7) $$\begin{array}{c} \text{Loss}=\frac{1}{N}\sum_{i=1}^{n}{\left\|P-Q\right\|}^{2}+1-\frac{\sigma \left(P,Q\right)}{\sigma \left(P\right)\times \sigma \left(Q\right)}. \end{array}$$

In Section 3.3, we quantitatively justify the rationale behind selecting the loss combination by comparing our results with those obtained using one evaluation criterion or two evaluation criteria as a loss function.

### 2.5. Implementation Detail

#### 2.5.1. Data Processing

We randomly sampled 70%, 10%, and 20% of all RGB-D images as the training, verification, and test sets, respectively.

Prior to data entry, the image size was adjusted to 224 × 224 pixels. To boost the generalization performance of the network, each RGB image was mean centered and normalized to unit variance using precomputed parameters before it was input to the network.

#### 2.5.2. Training Methods

The proposed HMAF method was trained by loading the weights of a pretrained VGG-16 network as initial weights. Eight RGB-D images were used during each iteration. The learning rate was initialized at 1 × 10^–4^. The values of the model parameters were learned via backpropagation of the loss function described in (6) using RMSprop. An early termination method was used to prevent model overfitting. Furthermore, we applied a transition of 0–2 pixels and random horizontal flips on both axes of the input RGB-D images to augment the dataset at training time. All experiments were conducted on a workstation equipped with an NVIDIA TITAN V GPU and 12 GB of RAM.

## 3. Experimental Results and Analysis

### 3.1. Datasets

To verify the prediction performance of our proposed HMAF network, all saliency prediction methods were applied to various datasets. Presently, there are very few public RGB-D datasets for performing visual saliency prediction research. This study evaluates the performance of saliency-prediction methods applied to two representative datasets—NUS3D [35] and NCTU [36], as follows: (1) NUS3D contains 600 RGB-D images and involves several 2D and RGB-D view scenes; it provides depth images, RGB stimuli, and 2D and RGB-D fixation maps; (2) NCTU comprises 475 RGB-D images along with depth maps; this dataset includes various scenes, most of which have been adapted from existing stereo movies and videos.

### 3.2. Evaluation Criteria

Four commonly used performance criteria—CC [20], Kullback–Leibler divergence [19] (KLDiv), area under the ROC curve [18] (AUC), and normalized scanpath saliency [22] (NSS)—were used to evaluate the prediction performance of the competing saliency-prediction methods [18, 19].

### 3.3. Comparison between Different Loss Functions

Table 1 compares single (KLDiv, CC′, and MSE) and combined (MSE + CC′, MSE + KLDiv, and CC′ + KLDiv) loss-function values. As seen from the table, the proposed method, on average, achieves better results than extant methods. The values of the combined loss functions, however, attain better results in favor of the proposed method compared to those of single functions. Our combined loss (MSE + CC′) obtains competitive prediction results on all criteria, unlike other loss functions. Based on this, all results were obtained by training the proposed HMAF with our combination.

### 3.4. Ablation Studies

#### 3.4.1. Effects of Hierarchical Features

To demonstrate the effects of hierarchical features, the output features from convolution layers Pool3, Pool4, and Pool5 were used for visual saliency prediction (we denoted this as models A, B, and C, respectively). Thus, hierarchical features can be considered important and complementary to each other, thereby achieving high-accuracy saliency prediction.

#### 3.4.2. Effect of VGGNet as Model Backbone

To further verify the effectiveness of the VGGNet, we kept the pipeline unchanged while only replacing the VGGNet with the ResNet block [37] (we denoted this as model D). The results are listed in Table 2. We can observe that applying the VGGNet-based approach generally provides better performance than the ResNet-based approach.

### 3.5. Comparison against State-of-the-Art

To demonstrate the effectiveness of the proposed HMAF-based saliency-prediction method, it was compared against four state-of-the-art approaches—Fang [24], DeepFix [19], DVA [20], SAM [21], EML [22], and SMI [23].  Table 3 lists the results of the quantitative comparison obtained by applying the competing methods to both the aforementioned datasets in terms of CC, KLDiv, AUC, and NSS. The results in Table 3 show that the proposed HMAF-based method clearly outperformed all others considerably, thereby demonstrating its superior effectiveness, robustness, and generalization capabilities.

**Figure 5:**
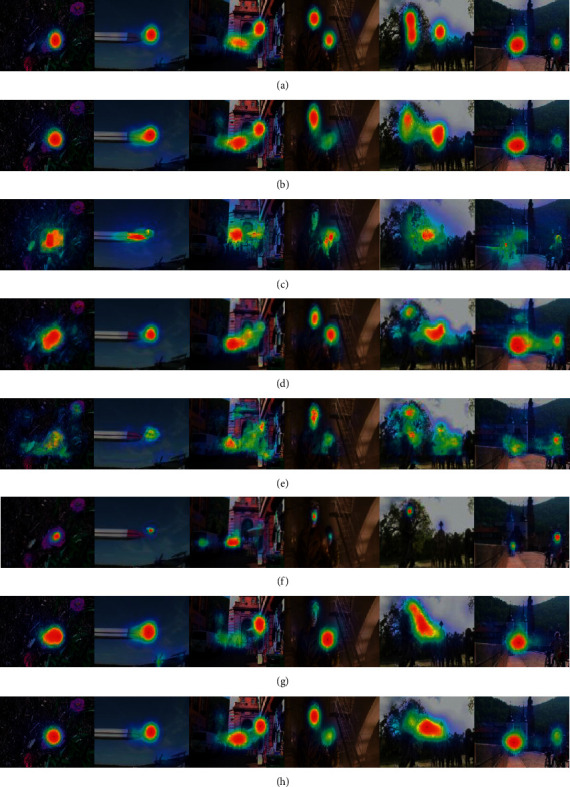
Qualitative results. (a) Ground-truth, (b) Proposed, (c) Fang, (d) DeepFix, (e) DVA, (f) SAM, (g) EML, and (h) SMI.

A qualitative comparison of the proposed HMAF method against the other six methods is depicted in Figure 5. Clearly, the proposed HMAF yields more accurate prediction results than other techniques because the saliency maps are fused by three two-input attention modules and a three-input attention module to facilitate high-accuracy prediction. It can be seen from Figure 5 that the proposed method is less distracted by high-contrast edges and complex backgrounds and that it can easily predict bottom-up saliency maps while also dealing well with global and local contrast levels. Another important fact is that the proposed method can highlight many top-down factors, such as human faces, people in complex backgrounds, and objects located at long and short distances from the camera. More specifically, although the images in lines 3, 4, 5, and 6 include complex backgrounds and various attention-grabbing objects, our proposed method can still highlight semantic regions preferentially.

Thus, the qualitative and quantitative experimental results both show that HMAF outperforms all other presently available techniques in terms of both robustness and accuracy.

### 3.6. Failure Case Analysis

**Figure 6:**
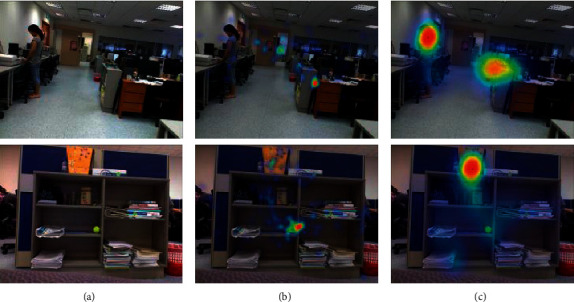
Some failure cases. (a) RGB. (b) Ground-truth. (c) Proposed.

Some typical failure cases are shown in Figure 6. The first row indicates that the proposed HMAF method does not perform well for RGB-D images with people. The second row indicates that when there are many objects, the proposed HMAF method prefers high-contrast objects and ignores small objects. To address this problem, we intend to consider deep networks [38, 39] to improve the performance of HMAF in the future.

### 3.7. Computational Complexity

The computational complexity of the proposed HMAF-based approach and other approaches was estimated from tests on the NCTU dataset. It takes approximately 1 h to train the proposed HMAF using an NVIDIA TITAN V GPU and an Intel i5-8500 3.0 GHz CPU. Inference takes approximately 0.01 s using the proposed HMAF on an image of size 640 × 480. In conclusion, our approach has low computational complexity and can satisfy the requirements of real-time image processing systems.

## 4. Conclusion

In this study, multimodal fusion of 3D data was studied, and a layered multimodal adaptive fusion network based on an attention module was proposed. The proposed network effectively extracts and combines features from different modes and levels. The dual input attention module uses the weights of importance associated with RGB and depth modes. Adaptive fusion of hierarchical features was extracted from the two patterns, rather than simply joining them together. In addition, the three-input attention module assigns different weights to the fusion significance characteristics at different levels for the RGB-D significance prediction. Experimental results show that the RGB-D significance prediction method based on HMAF is superior to all other advanced methods. This model exhibits superior performance, largely because of its attention mechanism design. It has the potential to mimic human visual systems more closely, which we hope to investigate in the future by introducing this technology to develop a direct convolution kernel that adapts the convolution kernel to identify targets quickly, allowing significant compression of the model parameters, for a variety of tasks. In addition, in the bottom-up process, if a feature refinement unit module can be designed for feature enhancement, the prediction errors in feature coding can be further repaired through the prior knowledge learned.

## Figures and Tables

**Table 1:** Comparison of quantitative scores of different loss function on two datasets.

Datasets	Criteria	MSE	KL	CC′	MSE + CC′	MSE + KL	CC′ + KL
NUS	CC	0.5224	0.5363	0.5296	**0.5533**	0.5473	0.5452
	KLDiv	1.1690	1.2344	1.1834	**1.0794**	1.1907	1.1320
	AUC	0.8519	**0.8637**	0.8627	0.8422	0.8571	0.8613
	NSS	2.0926	2.1698	2.1259	**2.3069**	2.2391	2.2086
NCTU	CC	0.8256	0.8390	0.8287	**0.8472**	0.8345	0.8308
	KLDiv	0.3176	**0.2736**	0.4877	0.3228	0.3333	0.3438
	AUC	0.9025	0.9087	0.9011	**0.9130**	0.9044	0.9041
	NSS	2.2957	2.3137	2.3199	**2.3493**	2.3437	2.3111

**Table 2:** Ablation analysis.

Datasets	Criteria	Model A	Model B	Model C	Model D	Proposed
NUS	CC	0.4889	0.5248	0.5295	0.5513	0.5533
	KLDiv	1.3543	1.2170	1.1741	1.1206	1.0794
	AUC	0.8440	0.8598	0.8693	0.8774	0.8422
	NSS	1.9523	2.1214	2.0917	2.0098	2.3069
NCTU	CC	0.7137	0.7941	0.8192	0.8364	0.8472
	KLDiv	0.4594	0.3235	0.3098	0.2926	0.3228
	AUC	0.8626	0.8997	0.9031	0.9105	0.9130
	NSS	1.9539	2.1786	2.3057	2.3070	2.3493

**Table 3:** The evaluation results of various saliency prediction models.

Datasets	Criteria	Fang	DeepFix	DVA	SAM	EML	SMI	Proposed
NUS	CC	0.333	0.4322	0.4549	0.5013	0.4857	0.5368	**0.5533**
	KLDiv	1.560	1.8138	2.4349	2.9059	2.2353	1.2501	**1.0794**
	AUC	0.795	0.7699	0.7236	0.7461	0.8149	0.8648	**0.8422**
	NSS	1.209	1.6608	1.7962	2.1259	1.9419	2.1843	**2.3069**
NCTU	CC	0.542	0.7974	0.6834	0.7115	0.7556	0.8376	**0.8472**
	KLDiv	0.674	1.3083	1.1045	1.8482	0.3886	0.4826	**0.3228**
	AUC	0.806	0.8650	0.8035	0.7250	0.8818	0.9053	**0.9130**
	NSS	1.264	1.8575	1.5546	1.5386	2.0666	2.3121	**2.3493**

## Data Availability

NUS3D data are available at https://sites.google.com/site/vantam/nus3d-saliency-dataset. NCTU are available at http://shallowdown.wixsite.com/chih-yao-ma/nctu-3dfixation-database.
